# Reliability and reproducibility of classification of distal ulnar fractures using cone beam computed tomography (CBCT)

**DOI:** 10.2340/17453674.2026.46344

**Published:** 2026-08-05

**Authors:** Linnéa WRETÖ, Lotta FORNANDER, Nina KÄMMERLING, Erika NYMAN, Maria MOLONEY

**Affiliations:** 1 Department of Biomedical and Clinical Sciences, Linköping University, Linköping; 2 Clinical Department of Orthopaedics in Norrköping, Region Östergötland, Norrköping; 3 Department of Health, Medicine and Caring Sciences, Linköping University, Linköping; 4 Clinical Department of Radiology in Motala, Region Östergötland, Motala; 5 Department of Orthopedic and Hand Surgery, Faculty of Medicine and Health, Örebro University, Örebro; 6 Clinical Department of Hand Surgery, Plastic Surgery and Burns in Linköping, Region Östergötland, Linköping, Sweden

## Abstract

**Background and purpose:**

Distal ulnar fractures (DUFs) remain under-investigated, primarily due to limited statistical power resulting from the low incidence. The present classification systems are difficult to use and are not validated for new radiological methods such as cone beam computed tomography (CBCT). We aimed to analyze the reliability and reproducibility of the 2 classification systems, Arbeitsgemeinschaft für Osteosynthesefragen foundation/Orthopaedic Trauma Association (AO/OTA) and Biyani, for fractures of the distal ulnar head and neck using CBCT images.

**Methods:**

76 patients with DUF who underwent a CBCT scan during the years 2016–2022 were included retrospectively. The CBCT images were reviewed twice by 6 observers of different medical specialties and levels of experience. All fractures were classified according to AO/OTA and Biyani. The reliability and reproducibility of each classification system were analyzed using kappa value analysis.

**Results:**

The reliability was fair to moderate for AO/OTA, Light’s ĸ 0.41 (95% confidence interval [CI] 0.38–0.45) and moderate for Biyani, Light’s ĸ 0.45 (CI 0.42–0.49), and the reproducibility was substantial for both AO/OTA, mean ĸ 0.65 (CI 0.49–0.80), and Biyani, ĸ 0.69 (CI 0.60–0.79).

**Conclusion:**

Both existing classification systems were perceived to be difficult to use and do not match existing fracture patterns. Using CBCT images, they both showed substantial reproducibility but not satisfactory reliability for clinical use.

When sustaining distal ulnar fractures (DUFs), older patients most often fracture the ulnar styloid as a result of low-energy falls, while younger patients more often suffer from ulnar neck or head fractures due to high-energy trauma [1].

Radiographs are traditionally used to classify DUFs and evaluate treatment [2–6]. Standard projections often include an anteroposterior and a lateral projection, where the latter provides limited information concerning the ulna, considering the over-projection of the radius. Cone beam computed tomography (CBCT) has been introduced as an alternative to radiographs [7,8] of extremities, with a lower radiation dose compared with regular CT [8,9] and marginally higher than the radiation dose of a series of radiographs [9]. Using CBCT provides more detailed imaging and enables 3D reconstruction, offering greater reliability than radiographs in assessing important fracture characteristics and guiding treatment in distal radial fractures (DRFs) [10,11]. However, the value of CBCT is yet undetermined for DUFs.

For a classification system to be clinically relevant, it must be reliable, reproducible, and easy to use in a clinical setting. The 2 main classification systems for DUFs today are Arbeitsgemeinschaft für Osteosynthesefragen foundation/Orthopaedic Trauma Association (AO/OTA) [12] and Biyani [13], which have shown poor reliability and reproducibility when using radiographs [6].

The aim of our study is to analyze the reliability and reproducibility of the 2 classification systems, AO/OTA and Biyani, for fractures of the distal ulnar head and neck using CBCT images.

## Methods

### Study design

Data was collected for all patients with a DUF with or without a concomitant radius fracture treated at the Vrinnevi Hospital, Norrköping, Sweden. It is a regional hospital that, since 2016, has used CBCT as the routine radiological method for all wrist and carpal bone trauma. Patients were consecutively and retrospectively identified by international classification of diseases (ICD) codes, in the digital patient register in Region Östergötland, for diagnoses of fractures of the distal and mid forearm (S522, S5220, S5221, S5240, S5241, S525, S5250, S5251, S526, S5260, S5261, S5270, S5271, S5280, S5281, S529, S5290). The images of these cases were reviewed to identify the distal ulnar head and neck fractures. The inclusion criteria were patients 18 years and older at the time of injury, with a fracture of the distal third of the ulna, and who underwent a CBCT examination before closed reduction at Vrinnevi Hospital during the years 2016–2022. Patients younger than 18 years or those with solely a fracture of the ulnar styloid, or with radiographs or conventional CT images instead of CBCT, were excluded. The images were pseudonymized, and no clinical information was provided. The GRRAS reporting guideline was used in the preparation of this article.

### Review process

6 observers independently reviewed and classified the fractures according to Biyani and AO/OTA. All observers were provided with the same written classification guide, with graphic descriptions of both classification systems (Appendix 1, see Supplementary data). The reviewers received no pre-review training or calibration before the classification process. Each review process was conducted within 1 week and then repeated after 2 months (±2 weeks) by all observers. The observers were 3 surgeons and 3 radiologists: a consultant in orthopedic surgery with extra interest in the upper extremity (LF, observer 1), a consultant in hand surgery (MM, observer 2) with 17 and 14 years of experience, respectively, a junior resident in orthopedics (LW, observer 3), 2 consultant radiologists (NK observer 5, JK observer 4) with 20 and 45 years of experience, respectively, and a fourth-year radiology resident (SC, observer 6). The images were presented as axial, coronal, and sagittal reconstructions. The slice thickness ranged from 0.2 mm to 3 mm, and the majority (n = 63) of cases had a slice thickness of 1.4 mm or less. The images were reviewed in ImageJ (U.S. National Institutes of Health, Bethesda, MD, USA), an image display software [14]. The observers were allowed to pan and zoom the images and adjust the window level to their preferences.

### Classifications

**Figure 1:**
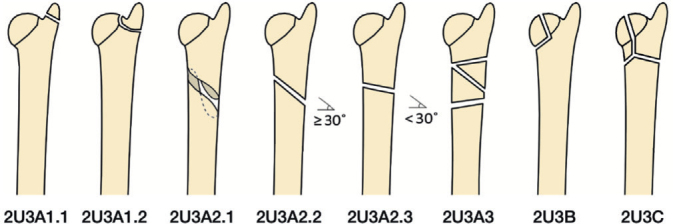
Arbeitsgemeinschaft für Osteosynthesefragen foundation/Orthopaedic Trauma Association (AO/OTA) classification for distal ulnar fractures 2018 [6,12].

**Figure 2:**
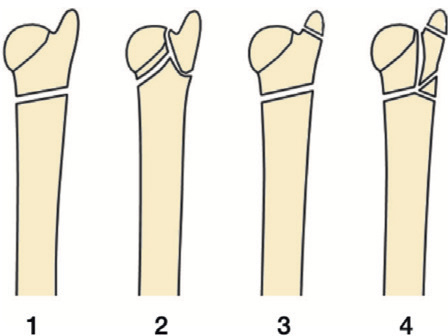
Biyani classification for distal ulnar fractures 1995 [6,13].

The AO/OTA classification [12] is a well-known and widely used system for classifying long-bone fractures. Since its first publication in 1996, it has been revised twice, in 2007 and in 2018. In the latter version, fractures of the distal ulna are classified irrespective of distal radius fractures. The AO/OTA classification for DUFs consists of 4 main groups, 1 of which consists of isolated styloid fractures, thus excluded from this study, and for the extraarticular fractures there are 4 subgroups (Figure 1). The Biyani classification [13] was published in 1995, describing fractures of the distal ulnar metaphysis in association with DRFs, with or without fractures of the ulnar styloid. It consists of 4 types of fractures (Figure 2).

### Statistics

Categorical data is presented as numbers (%) and analyzed using the chi-square test (Fisher’s exact test if n < 5 in a group). Continuous data is presented as median (interquartile range, 25th to 75th percentiles [IQR]). Cohen’s kappa value was used to determine both the intra- (reproducibility) and inter-rater agreement (reliability), and the inter-rater agreement [15] was computed as the mean of all observer-pairs [15,16]. All kappa values (ĸ) and means are presented with 95% confidence intervals (CI). The criteria of Landis and Koch were used to interpret the kappa values [17]. The level of significant agreement was set to substantial, ĸ 0.61–0.80. The extent of missing data is expected to be limited and unlikely to impact the results significantly. IBM SPSS Statistics for Macintosh, version 29.0.0.0 (IBM Corp Armonk, NY, USA) was used for all statistical analyses.

### Ethics, funding, and potential conflicts of interest

The study protocol was approved by the Swedish ethical approval authority (DNR: 2023-01189-01). This work was supported by ALF Grants (register numbers RÖ-978765, RÖ-1005512, and RÖ-1012105), Region Östergötland, Sweden; RALF Grants (06001113) and Sinnescentrum Grants (RÖ-2024-3675-011), Region Östergötland, Sweden. The authors declare no conflicts of interest. Complete disclosure of interest forms according to ICMJE are available on the article page, doi: 10.2340/17453674.2026.46344

## Results

**Figure 3:**
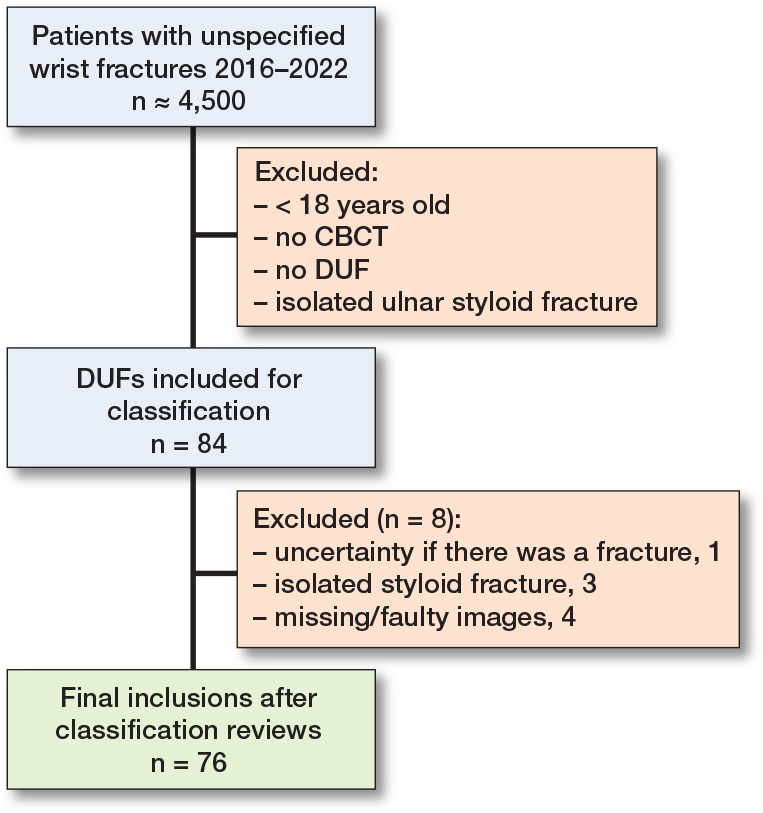
Flowchart of the inclusion process showing identification of relevant distal ulnar fractures (DUFs) among patients with unspecified wrist fractures diagnosed by cone beam computed tomography (CBCT).

**Figure 4:**
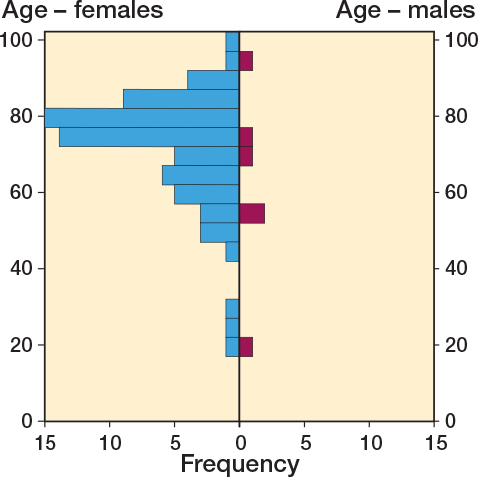
Population pyramid showing age distribution by sex among patients with distal ulnar fractures

76 patients were finally included after the 2 review sessions conducted by all 6 observers; 8 patients were thereafter excluded because they did not meet the inclusion criteria (Figure 3). Age was normally distributed among females but not among males. At the time of injury, the median age was 75 years (IQR 62–81). There were significantly more females (n = 70, 92%), than males (n = 6, 8%), and the median age was 75 (IQR 62–81) and 63 (IQR 45–78) years, respectively (Figure 4, Table 1). The right wrist was fractured in 31 (41%) patients and the left in 45 (59%). A concomitant DRF was identified in 65 (86%) patients (Table 1). Missing values were found in 9 patients. In 6 of these, 2 individual reviewers considered exclusion appropriate due to movement artefacts (1 reviewer in 1 case on both review occasions) or because the fracture was located at the border of the styloid base (1 reviewer in 2 cases on both review occasions). In the remaining 3 cases, the missing data resulted from misclassification, where AO/OTA codes were incorrectly reported in the Biyani column or vice versa.

**Table 1:** Demographics of included patients with distal ulnar fractures (DUF) with or without a distal radial fracture (DRF)

Factor	Total	Female	Male
Patients, n (%)	76	70 (92)	6 (7.9)
Median age (IQR)	75 (62–81)	75 (62–81)	63 (45–78)
Fractured side (right/left)	31/45	27/43	4/2
Concomitant DRF/isolated DUF	65/11	61/9	4/2

IQR = interquartile range.

### Reliability and reproducibility

The intra-rater agreement was overall substantial for both AO/OTA, ĸ 0.65 (CI 0.49–0.80), and Biyani, ĸ 0.69 (CI 0.60–0.79) (Table 2). The inter-rater agreement for AO/OTA was moderate in total, ĸ 0.41 (CI 0.38–0.45), and in the 1st review, ĸ 0.45 (CI 0.41–0.49), and fair in the 2nd review, ĸ 0.38 (CI 0.24–0.40) (Table 3). Further, the inter-rater agreement for Biyani was moderate in total, ĸ 0.45 (CI 0.42–0.49), 1st ĸ 0.44 (CI 0.39–0.49), and 2nd review, ĸ 0.47 (CI 0.40–0.53) (Table 3). All raw kappa values are presented in Appendices 2 and 3 (see Supplementary data).

**Table 2:** Outcomes of intra-rater agreement among 6 observers classifying distal ulnar fractures using the AO/OTA and Biyani classifications

Classification Observer	Intra-rater Kappa value (CI)	Agreement
AO/OTA		
1	0.66 (0.54–0.79)	Substantial
2	0.54 (0.41–0.67)	Moderate
3	0.70 (0.57–0.82)	Substantial
4	0.47 (0.33–0.60)	Moderate
5	0.91 (0.83–0.99)	Almost perfect
6	0.60 (0.46–0.74)	Moderate
Mean	0.65 (0.49–0.80)	Substantial
Biyani		
1	0.67 (0.55–0.80)	Substantial
2	0.61 (0.48–0.74)	Moderate ^**a**^
3	0.80 (0.69–0.91)	Substantial
4	0.60 (0.46–0.74)	Moderate
5	0.82 (0.72–0.92)	Almost perfect
6	0.66 (0.53–0.79)	Substantial
Mean	0.69 (0.60–0.79)	Substantial

Landis and Koch criteria: kappa; ≤ 0.2, slight agreement; 0.21–0.40, fair agreement; 0.41–0.60, moderate agreement; 0.61–0.80, substantial agreement; and ≥ 0.81, almost perfect agreement.

AO/OTA = Arbeitsgemeinschaft für Osteosynthesefragen/Orthopaedic Trauma Association; CI = 95% confidence interval.

a Original number 0.608, moderate

**Table 3:** Outcomes of inter-rater agreement among 6 observers classifying distal ulnar fractures using the AO/OTA and Biyani classifications

Classification Review	Inter-rater Light’s kappa (CI)	Agreement
AO/OTA		
1st	0.45 (0.41–0.49)	Moderate
2nd	0.38 (0.24–0.40)	Fair
Total	0.41 (0.38–0.45)	Moderate
Biyani		
1st review	0.44 (0.39–0.49)	Moderate
2nd review	0.47 (0.40–0.53)	Moderate
Total	0.45 (0.42–0.49)	Moderate

See footnote to Table 2.

There was no difference in kappa values between the surgeons and radiologists in either intra- or inter-rater agreement for both classification systems. However, the surgeons tended to demonstrate more consistent (i.e., similar) kappa values, whereas the radiologists exhibited a greater variability in agreement levels.

A common disagreement in the AO/OTA classification subgroups was between 2U3C (complete articular fracture) and 2U3A2.3 (extraarticular transverse fracture) (see Figure 1). However, the 2U3C group was also much agreed upon, suggesting that many fractures were comminuted and articular. In the Biyani classification, a common disagreement was between types 2 and 4, which have quite similar fracture patterns, but type 4 consists of more comminution of the ulnar metaphysis (see Figure 2). Biyani type 1, simple extraarticular fracture, was quite commonly agreed upon. The 2 classification systems are not directly comparable across fracture groups or subgroups, as the underlying fracture morphologies differ, leading to substantial variation in the fractures most commonly agreed upon.

## Discussion

We aimed to analyze the reliability and reproducibility of the 2 classification systems, AO/OTA and Biyani, for fractures of the distal ulnar head and neck using CBCT images. We found overall moderate reliability (inter-rater agreement) and substantial reproducibility (intra-rater agreement) for both the AO/OTA and the Biyani classification. While reproducibility was higher than reliability, clinical reliability is equally important to guarantee equitable care for the patient, irrespective of the clinician involved. The intra-rater agreement was very similar between the 2 classification systems for all observers, suggesting that neither AO/OTA nor Biyani was superior. Although half of the observers perceived Biyani to be easier to use, the remaining 50% thought the classifications were equally difficult to use. This was mainly described as the fracture types in Biyani being more consistent with the actual fractures, unlike AO/OTA, and that there were fewer groups/subgroups, making it more convenient to use. This might be explained by the development of the Biyani classification, which was based on a series of actual fractures (although quite few in numbers, only 18 patients), compared with AO/OTA, which was systematically developed for commonly occurring fractures of the long bones in the body and not specific to DUF patterns.

Furthermore, the intra-rater agreement showed substantial agreement between the observers for both classification systems. Intra-rater agreement was similar between radiologists and surgeons; the surgeons showed more consistent kappa values, whereas the radiologists showed greater variation among themselves in the AO/OTA but not in the Biyani classification. This difference may be explained by the surgeons being more familiar with the AO/OTA classification as it is more regularly used in their clinical work, predominantly for other fractures than those of the ulna. Although the AO/OTA classification is scarcely ever used for DUFs in clinical practice, except for registration in the Swedish fracture register, it has no clear connection to the choice of treatment. To account for potential observer bias, we selected observers from different specialties and levels of experience to represent doctors who encounter patients with DUFs in their day-to-day work.

Regarding inter-rater agreement of AO/OTA, there was no improvement from the 1st to 2nd review, rather a decrease. In contrast, inter-rater agreement for Biyani showed slightly better results in the 2nd review compared with the 1st, which might suggest that Biyani was easier to use and comprehend in the 2nd review. Further development of the Biyani classification may result in a more comprehensive classification, applicable in clinical practice for DUFs with or without a concomitant DRF.

There were significantly more females in the study population, which is a common feature in studies of wrist fractures, as they are highly related to osteoporosis/osteopenia. Although the sex skew is particularly high in this study (92% females) compared with other studies with similar populations of 76% [1], 82% [6], 89% [18], 92% [4], females, which could indicate that DUFs are even more related to osteoporosis/osteopenia than DRFs. The limited representation of male patients in this study may restrict the ability to draw reliable conclusions regarding DUFs in the male population.

DUFs are uncommon, which limits the ability to study them in larger populations. Furthermore, this study is also constrained to data collected from 2016 onwards, as CBCT was not implemented in our clinical practice before that. In contrast to previous studies, mostly regarding treatment of DUFs, which have been limited to small populations of about 20–30 patients, our population of 76 patients was quite large. This population was extracted from a large group of about 4,500 patients receiving care for any wrist fracture, thus making selection bias unlikely.

To evaluate the review process, a questionnaire was sent to and answered by the observers, and all were fairly unanimous that not all fracture patterns are covered by AO/OTA and Biyani, which enabled individual interpretation of the classification groups/subgroups. Each observer likely interpreted both classification systems similarly in the 1st and 2nd reviews, as indicated by the substantial reproducibility. In addition, the time interval between reviews was sufficiently long to forget the fractures, reducing the likelihood of recall bias. However, the observers might have had a different propensity to “force” fractures into groups instead of leaving them as “could not be classified”. Within the AO/OTA classification system, distinguishing between oblique (2U3A2.2) and spiral (2U3A2.1) fractures proved particularly challenging, and it remains unclear whether this distinction is of any clinical relevance. Fracture types 2 and 4 in the Biyani classification were also found challenging to distinguish, as both involve articular engagement and metaphyseal comminution. However, type 4 appears to exhibit greater comminution, which can be difficult to accurately identify in actual fractures. The Biyani classification is specifically focused on fractures of the distal ulnar metaphysis and does not include fractures of the distal diaphysis. In contrast, the AO/OTA classification was perceived to lack a fracture group with great comminution and articular engagement.

In 7 cases, images of a 3D reconstruction of the fracture were available; not all reviewers took this into account when classifying the fractures, but some did, which might have influenced their judgement. In some cases, the 3D reconstruction confirmed the classification made in the 2D review, but in others it provided a more detailed overall picture, prompting reviewers to revise their assessments. Moloney et al. studied the reliability and reproducibility of AO/OTA and Biyani for DUFs, using plain radiographs, and found moderate reliability and reproducibility for all classification systems [6]. A topic described by Moloney et al. to be difficult when reviewing radiographs was articular engagement, and the authors suggested that this could be better assessed by, for example, CBCT [6]. It was not perceived by the observers in our study to be difficult to assess articular engagement in DUFs using CBCT images, thus suggesting that CBCT should be used for mapping and classification of DUFs. Furthermore, based on the present results, it seems that the reproducibility is slightly higher when using CBCT images compared with radiographs, and we therefore recommend CBCT for the classification and mapping of DUFs. However, present classifications are based on DUFs visualized by radiographs and not CBCT. Possibly, a new classification system based on common DUF patterns in CBCT images could lead to higher accuracy, reliability, reproducibility, and perhaps, in the long term, a classification-based treatment algorithm.

Eriksson et al. [11] compared radiographs and CBCT in DRFs and 2 classification systems, AO/OTA and Buttazzoni, and showed that an additional CBCT increased the inter-rater agreement in AO/OTA, and was unchanged in Buttazzoni [11]. In line with the results of the present study and the study of Moloney et al. [6], there was no change in inter-rater agreement when comparing fracture classification in radiographs and CBCT. Eriksson et al. also showed that the classification groups/types changed from simple, extra-articular fractures in the radiographs to more advanced and intra-articular fractures when reviewing the CBCT images, and a change in treatment approach was observed in 38% of cases following the addition of CBCT imaging [11]. These changes occurred in both directions, from less to more advanced treatment and vice versa.

### Limitations

The retrospective design of this study comes with both pros and cons; it enabled a larger population but limited control over the quality and consistency of radiographic imaging, potentially introducing variability in interpretation. A common limitation in evaluating classification systems with multiple subgroups is the need for a large sample size to ensure sufficient subgroup representation for reliable statistical results. In this study, however, kappa statistics were calculated for the overall classification systems rather than by subgroup, which mitigates this limitation.

### Conclusion

Our study demonstrates that the reliability of AO/OTA and Biyani was moderate when using CBCT images, and comparable to plain radiographs. The reproducibility was substantial and slightly greater compared with plain radiographs, but the classification systems were perceived as difficult to use, especially AO/OTA, suggesting that they are not sufficient for easy use in clinical practice.

*In perspective,* we encourage further research into the refinement of the classification systems to reflect the enhanced detail of the DUF pattern provided by CBCT, which in future research might contribute to a foundation for a treatment algorithm for DUFs.

### Supplementary data

Classification guide and individual inter-rater results are available as Supplementary data on the article home page, doi: 10.2340/17453674.2026.46344

## Supplementary Material



## Notes

Handling co-editors: Jeppe Vejlgaard Rasmussen and Robin Christensen

*Acta* thanks Tokutake Katsuhiro and Morten Kjaer for help with peer review of this manuscript.

The study was designed by LW, LF, EN, and MM. All authors, except EN, participated as observers in the fracture classification. LW was responsible for data collection, analysis, and manuscript drafting. LF, NK, EN, and MM contributed to data analysis and critically revised the manuscript. All authors reviewed and approved the final version prior to submission.

The authors would like to acknowledge Jan Kåredal and Salma Choura for participating as observers in CBCT reviews, John Arfvidsson for reviewing all radiographs and filtering out the DUFs, and Mats Fredrikson and Lars Valter for their support with the statistical analysis.
