# Supplementary material for: Reliability and reproducibility of classification of distal ulnar fractures using cone beam computed tomography (CBCT)
Source: Acta Orthop. 2026 Aug 5;97:517–22. doi: 10.2340/17453674.2026.46344 (PMC13444841; doi:10.2340/17453674.2026.46344)
Supplement: Supplementary file 1 [file ActaO-97-46344-s1.pdf]

Supplementary data

Appendix 1: Classification guide

Classification Guide Ulnar Fractures

Add your name and number of review (1 or 2) in the file name when you save and send the excel document in. Also add name, number of review and start- and finish date for the review at the top of the document before you send it to the given e-mail address.

AO/OTA classification

Main group: A, B or C translates to 1, 2 and 3. Register 1, 2 or 3 and if 1 see below.
Classify according to the descriptive text and register the corresponding bold number.

Ulna, distal end segment,
extraarticular fracture
2U3A

1

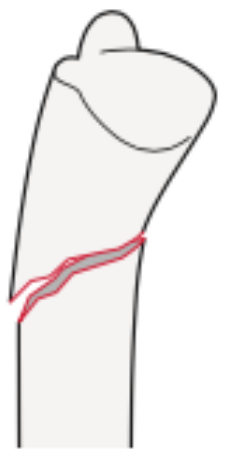

Ulna, distal end segment,
partial articular fracture
2U3B

2

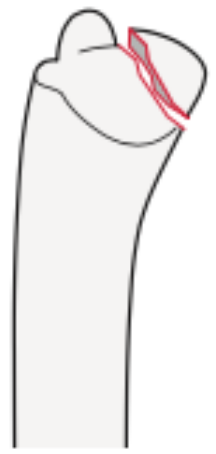

Ulna, distal end segment,
complete articular fracture
2U3C

3

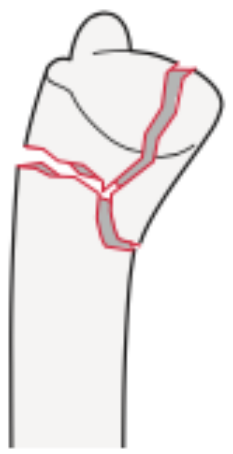

If type A/1-fracture, extra-articular fracture, classify according to sub-groups: classify according to the descriptive text and register the corresponding bold number(first number is main group A=1 and remaining numbers as of sub-group e.g. (2U3) A2.1=121)

Group: Ulna, distal end segment, extraarticular, **simple fracture** 2U3A2

Subgroups:

Spiral fracture
2U3A2.1

121

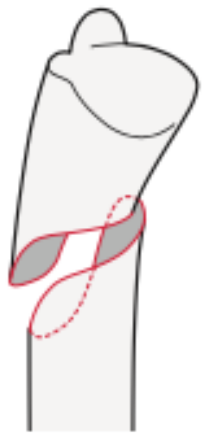

Oblique fracture (≥30°)
2U3A2.2

122

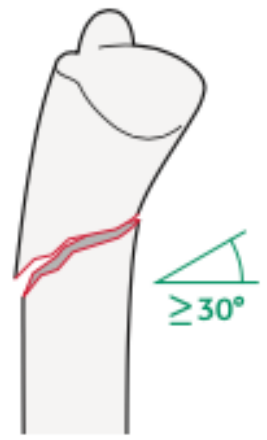

Transverse fracture (<30°)
2U3A2.3

123

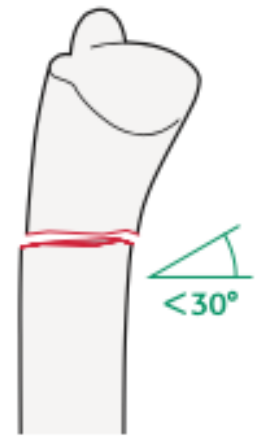

Group: Ulna, distal end segment, extraarticular, **multifragmentary fracture** 2U3A3

13

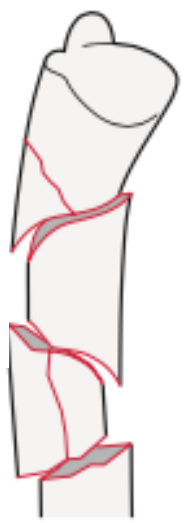

Biyani classification:

Classify according to the description for type 1-4 and register the corresponding number.

A 1

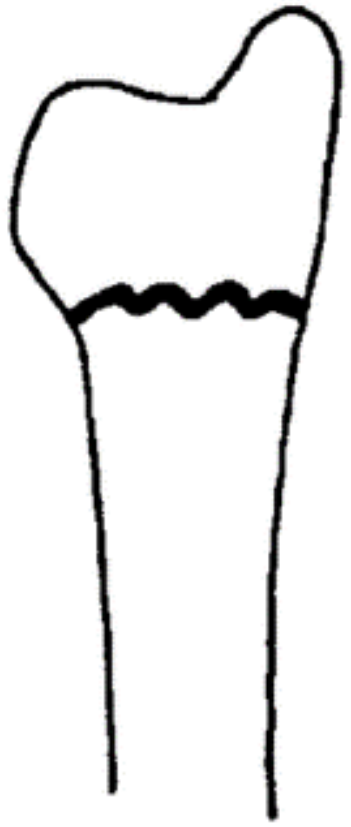

Type 1: Extra-articular fractures with minimal styloid

2

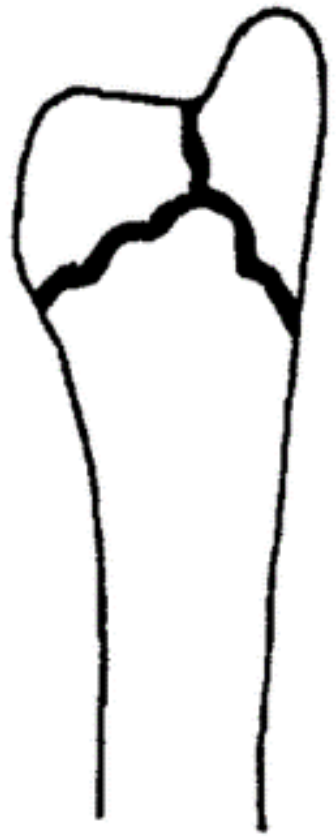

Type 2: Fractures of the metaphysis and the ulnar comminution

3

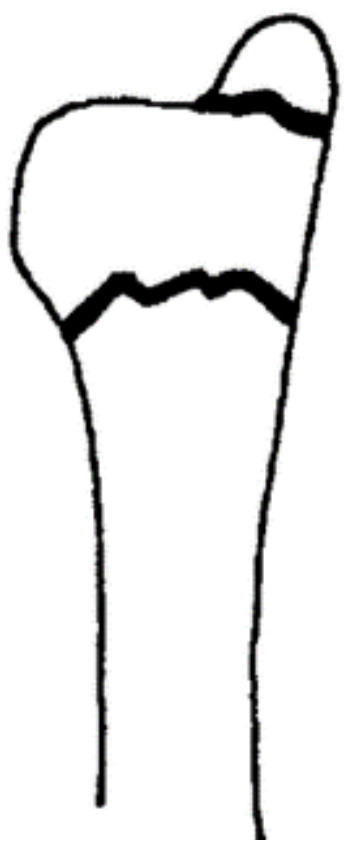

4

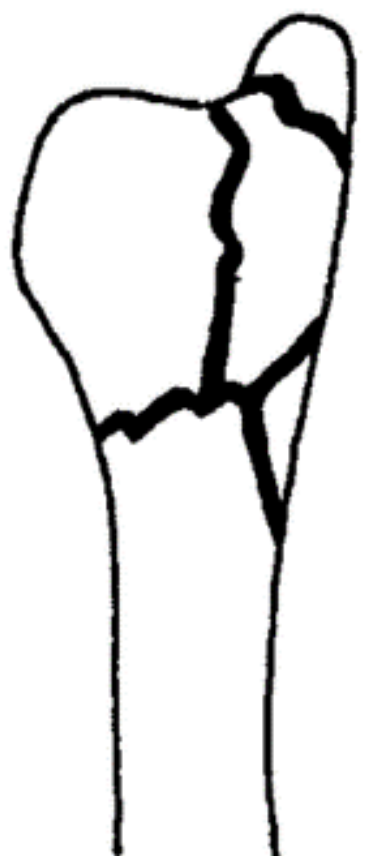

Type 3: Ulna fracture combined with fracture ulna metaphysis

Type 4: Completely comminuted fracture of the of the ulnar styloid

If the fracture does not match any of the fracture types, or groups in the classification systems:
Register a 9 in AO/OTA or Biyani and a 1 to the right in the intended column for each classification system that cannot be classified, se excel file. Comment if the fracture is too proximal(not within the distal third of the ulna) or if it is an isolated styloid fracture since these need to be excluded.

Appendix 2: Inter-rater results of each observer-pairs for AO/OTA

Inter-rater AO/OTA	Observers	Kappa	95% CI	Agreement
1st review	1 vs 2	0.558	0.429 to 0.687	Moderate
	1 vs 3	0.537	0.398 to 0.676	Moderate
	1 vs 4	0.431	0.300 to 0.562	Moderate
	1 vs 5	0.537	0.404 to 0.670	Moderate
	1 vs 6	0.438	0.311 to 0.565	Moderate
	2 vs 3	0.487	0.354 to 0.620	Moderate
	2 vs 4	0.383	0.250 to 0.516	Fair
	2 vs 5	0.451	0.322 to 0.580	Moderate
	2 vs 6	0.284	0.168 to 0.400	Fair
	3 vs 4	0.417	0.286 to 0.548	Moderate
	3 vs 5	0.459	0.314 to 0.604	Moderate
	3 vs 6	0.432	0.301 to 0.563	Moderate
	4 vs 5	0.490	0.359 to 0.621	Moderate
	4 vs 6	0.341	0.214 to 0.468	Fair
	5 vs 6	0.470	0.349 to 0.592	Moderate
	Light´s Kappa	0.448	0.409 to 0.487	Moderate
2nd review	1 vs 2	0.392	0.253 to 0.531	Fair
	1 vs 3	0.549	0.420 to 0.678	Moderate
	1 vs 4	0.263	0.157 to 0.369	Fair
	1 vs 5	0.494	0.359 to 0.629	Moderate
	1 vs 6	0.408	0.277 to 0.539	Fair
	2 vs 3	0.452	0.315 to 0.589	Moderate
	2 vs 4	0.248	0.140 to 0.356	Fair
	2 vs 5	0.468	0.331 to 0.605	Moderate

	2 vs 6	0.332	0.205 to 0.459	Fair
	3 vs 4	0.308	0.192 to 0.424	Fair
	3 vs 5	0.659	0.524 to 0.794	Substantial
	3 vs 6	0.354	0.225 to 0.483	Fair
	4 vs 5	0.317	0.203 to 0.431	Fair
	4 vs 6	0.098	0.014 to 0.182	Slight
	5 vs 6	0.374	0.241 to 0.507	Fair
	Light´s Kappa	0.381	0.241 to 0.395	Fair
Total mean		0.414	0.375 to 0.453	Moderate

Appendix 3: Inter-rater results of each observer-pairs for Biyani

Inter-rater Biyani	Observers	Kappa	95% CI	Agreement
1st review	1 vs 2	0.495	0.364 to 0.626	Moderate
	1 vs 3	0.444	0.303 to 0.585	Moderate
	1 vs 4	0.410	0.271 to 0.549	Moderate
	1 vs 5	0.452	0.313 to 0.591	Moderate
	1 vs 6	0.319	0.190 to 0.448	Fair
	2 vs 3	0.530	0.391 to 0.669	Moderate
	2 vs 4	0.560	0.425 to 0.695	Moderate
	2 vs 5	0.571	0.436 to 0.706	Moderate
	2 vs 6	0.365	0.240 to 0.490	Fair
	3 vs 4	0.433	0.290 to 0.576	Moderate
	3 vs 5	0.372	0.225 to 0.519	Fair
	3 vs 6	0.357	0.235 to 0.479	Fair
	4 vs 5	0.581	0.444 to 0.718	Moderate
	4 vs 6	0.307	0.191 to 0.423	Fair
	5 vs 6	0.415	0.290 to 0.540	Moderate
	Light´s Kappa	0.441	0.391 to 0.491	Moderate
2nd review	1 vs 2	0.517	0.382 to 0.652	Moderate
	1 vs 3	0.560	0.423 to 0.697	Moderate
	1 vs 4	0.472	0.333 to 0.611	Moderate
	1 vs 5	0.555	0.422 to 0.688	Moderate
	1 vs 6	0.263	0.136 to 0.390	Fair
	2 vs 3	0.550	0.413 to 0.687	Moderate
	2 vs 4	0.428	0.287 to 0.569	Moderate
	2 vs 5	0.627	0.498 to 0.756	Substantial

	2 vs 6	0.411	0.278 to 0.544	Moderate
	3 vs 4	0.430	0.283 to 0.577	Moderate
	3 vs 5	0.589	0.456 to 0.722	Moderate
	3 vs 6	0.379	0.252 to 0.506	Fair
	4 vs 5	0.472	0.335 to 0.609	Moderate
	4 vs 6	0.251	0.137 to 0.365	Fair
	5 vs 6	0.474	0.341 to 0.607	Moderate
	Light´s Kappa	0.465	0.404 to 0.526	Moderate
Total mean		0.453	0.417 to 0.489	Moderate
